# Raphidioptera of Canada

**DOI:** 10.3897/zookeys.819.26626

**Published:** 2019-01-24

**Authors:** David C.A. Blades

**Affiliations:** 1 Research Associate, Royal British Columbia Museum, 675 Belleville St, Victoria, BC, V8W 9W2, Canada Royal British Columbia Museum Victoria Canada

**Keywords:** biodiversity assessment, Biota of Canada, Raphidioptera, snakeflies

## Abstract

There are eight species in two families of Raphidioptera known from Canada, an increase of one species since the prior assessment in 1979. Another four species are likely to occur in Canada based on DNA evidence and distributional records. The Barcode of Life Data System currently lists ten Barcode Index Numbers for Canadian Raphidioptera.

Raphidioptera, commonly referred to as snakeflies, is a small order of insects containing two extant families: Raphidiidae and Inocelliidae. Species are confined to the northern hemisphere with the greatest diversity around the Mediterranean Sea and in eastern Asia. The North American species only occur west of the Rocky Mountains from about 53° N latitude south to Guatemala. North-central California is the area of greatest diversity in western North America (Aspöck and Aspöck 2014, Wu and Liu 2016). Members of both Raphidiidae (*Agulla*) and Inocelliidae (*Negha*) occur in Canada. All Canadian species are restricted to Pacific Maritime, Western Interior Basin, and Montane Cordillera ecozones south of 53° N in British Columbia and one, *Agullaadnixa* (Hagen), with a range extending eastward into the western edge of the Prairies ecozone in southern Alberta (Table 1). All Canadian species can be found across most of southern British Columbia with the exception of *Agullacrotchi* (Banks), found only in the Western Interior Basin ecozone. *Agullaadnixa* and *A.assimilis* (Albarda) are the most commonly collected and widespread species.

**Table 1.** Census of Raphidioptera in Canada.

Taxon^1^	No. species reported in Kevan (1979)	No. species currently known from Canada	No. BINs^2^ available for Canadian species	Est. no. undescribed or unrecorded species in Canada	General distribution by ecozone^3^	Information sources^4^
Raphidiidae	6	6	9	3	Pacific Maritime, Western Interior Basin, Montane Cordillera, Prairies	Penny et al. 1997, Scudder and Cannings 2009; specimens in UBC, RBCM, CNC, NFRC
Inocelliidae	1	2	1	1	Pacific Maritime, Western Interior Basin, Montane Cordillera	Penny et al. 1997, Scudder and Cannings 2009; specimens in UBC, RBCM, CNC
**Total**	**7**	**8**	**10**	**4**		

**^1^**Classification follows that indicated in Kevan (1979). **^2^**Barcode Index Number, as defined in Ratnasingham and Hebert (2013). ^3^See figure 1 in Langor (2019) for a map of ecozones. **^4^**UBC – University of British Columbia Spencer Entomological Collection, Vancouver, BC; RBCM – Royal British Columbia Museum, Victoria, BC; CNC – Canadian National Collection of Insects, Arachnids, and Nematodes, Ottawa, ON; NFRC – Northern Forestry Centre, Edmonton, AB.

Raphidioptera require a cold period to develop properly and pupate. Most species spend two years as larvae and have 10 to 15 instars. According to Carpenter (1936) habitat specificity of the larvae limits the range of snakeflies to forested portions of western North America; however, it is now known that only the Inocelliidae and a few Raphidiidae are restricted to the bark of trees as larvae while most other species can also be found on or under the soil surface feeding on small arthropods (Aspöck 2002, Aspöck et al. 2012a). Adult Raphidiidae are also predaceous on small insects whereas adults of Inocelliidae eat pollen or do not feed at all (Aspöck et al. 2012b). The lifespan of adult Inocelliidae is only a few days, a characteristic which may explain the relative rarity of the single genus *Negha* in museum collections in North America.

When the Raphidioptera of Canada were last assessed (Kevan 1979), there were 168 extant species (151 Raphidiidae; 17 Inocelliidae) known worldwide (Aspöck 1986). According to Aspöck (1986) there were certainly no more than 200 species. Approximately 30 years later, the number of described, extant species was 248 (206 Raphidiidae; 42 Inocelliidae) and the estimated total global fauna is nearly 300 species (Aspöck and Aspöck 2014, Engel et al. 2018). In that same period, the number of species known from North America decreased from 19 to 18 as a result of taxonomic revision (Kevan 1979, Penny et al. 1997).

According to the paleobiology database (https://fossilworks.org), the fossil record contains 119 species of Raphidioptera in eight families worldwide. In North America 15 species are listed in the fossil record database. Of the three known Canadian specimens, two Cretaceous Mesoraphidiidae were found in Alberta and Labrador and *Archiinocelliaoligoneura* Handlirsch (family uncertain) from the Paleogene in British Columbia (https://fossilworks.org; Aspöck et al. 2012a).

Very little research has been done on the snakeflies of North America since the 1970s (Penny et al. 1997, Aspöck and Aspöck 2014). This is reflected by the small change in number of species known from Canada over this period (Table 1). Kevan (1979) synonomised *Neghainflata* (Hagen) and *N.longicornis* (Albarda). These two *Negha* species are now regarded as distinct, resulting in an increase in known Inocelliidae (Penny et al. 1997; ITIS database: https://www.itis.gov; BOLD: http://www.boldsystems.org). Half of the known Canadian species have associated Barcode Index Numbers (BINs). Seven BINs of *Agulla* are not currently placed to species, which indicates that there are undescribed species in this taxon. It is likely that intensive collecting efforts and taxonomic revision of the group in North America will uncover at least a few new species and range extensions. It is estimated that an additional two species occur in Canada. If predictions of climate change are correct some southern species may be expected to eventually appear in Canada as their ranges shift northward.
